# Synchronization of the Glycolysis Reaction-Diffusion Model via Linear Control Law

**DOI:** 10.3390/e23111516

**Published:** 2021-11-15

**Authors:** Adel Ouannas, Iqbal M. Batiha, Stelios Bekiros, Jinping Liu, Hadi Jahanshahi, Ayman A. Aly, Abdulaziz H. Alghtani

**Affiliations:** 1 Laboratory of Dynamical Systems and Control, University of Larbi Ben M’hidi, Oum El Bouaghi 04000, Algeria; ouannas.adel@univ-oeb.dz; 2 Department of Mathematics, Faculty of Science and Technology, Irbid National University, Irbid 2600, Jordan; ibatiha@inu.edu.jo; 3 Nonlinear Dynamics Research Center (NDRC), Ajman University, Ajman 346, United Arab Emirates; 4 Department of Banking and Finance, FEMA, University of Malta, MSD 2080 Msida, Malta; 5 LSE Health, Department of Health Policy, London School of Economics and Political Science, London WC2A 2AE, UK; 6 Hunan Provincial Key Laboratory of Intelligent Computing and Language Information Processing, Hunan Normal University, Changsha 410081, China; Ljp202518@163.com; 7 Department of Mechanical Engineering, University of Manitoba, Winnipeg, MB R3T 5V6, Canada; jahanshahi.hadi90@gmail.com; 8 Department of Mechanical Engineering, College of Engineering, Taif University, P.O. Box 11099, Taif 21944, Saudi Arabia; aymanaly@tu.edu.sa (A.A.A.); a.ghtani@tu.edu.sa (A.H.A.)

**Keywords:** synchronization, linear control, asymptotic stability, reaction-diffusion model, lyapunov function, Selkov system, glycolysis system

## Abstract

The Selkov system, which is typically employed to model glycolysis phenomena, unveils some rich dynamics and some other complex formations in biochemical reactions. In the present work, the synchronization problem of the glycolysis reaction-diffusion model is handled and examined. In addition, a novel convenient control law is designed in a linear form and, on the other hand, the stability of the associated error system is demonstrated through utilizing a suitable Lyapunov function. To illustrate the applicability of the proposed schemes, several numerical simulations are performed in one- and two-spatial dimensions.

## 1. Introduction

The development of control laws to achieve synchronization is one of the most valuable aspects in the analysis of the different behaviors of natural systems. Synchronization is necessary to increase our knowledge of a wide variety of naturalistic problems designed to meet the needs of biological processes associated, for instance, with living cells and the neuronal network technology. Such schemes can also be efficiently employed to improve the power of lasers, to encode and decode electronic messages, and to establish secure methods of communication [1]. In recent years, the synchronization of one-dimensional equations has been extensively examined and is broadly well-understood. In this regard, many methods and schemes have been developed to achieve the synchronization of ordinary differential equations (ODEs) and discrete maps, including the linear or nonlinear control scheme, the adaptive control approach, the active control scheme, the feedback control method [2,3,4,5,6,7,8,9,10,11,12,13,14,15], and various other types of synchronization, which can be found in [16,17,18,19,20,21,22,23,24,25,26,27,28,29,30,31]. However, the research dealing with synchronizing spatially extended systems described by reaction-diffusion systems (RDSs) is still limited.

Differential equations have wide applications in various engineering and science disciplines [32,33]. In chemistry, several chemical reaction models are described in the form of differential equations. Glycolysis, a basic chemical reaction occurring in the cytosol, is considered to be the typical example of a metabolic pathway for cellular energy [34]. The abundance of glycolysis makes it one of the ancient metabolic pathways for providing energy via the breakdown of glucose $C_{6}H_{12}O_{6}$, into pyruvate, $CH_{3}COCOO^{-}+\;H^{+}$ [35]. The overall reaction of glycolysis is represented as follows [36]:

$$C_{6}H_{12}O_{6}+2NAD^{+}+2ADP+2P\to 2pyruvicacid,$$

$$(CH_{3}\left(C=O\right)COOH+2ATP+2NADH+2H^{+}).$$

Thus, glycolysis is a source of protons, and under increased oxygen-independent energy demands (during the exercise of muscles or cell proliferation), glycolysis may generate more protons and decrease the cytoplasmic pH. Selkov [37] presented a basic system that makes it possible to explain most experimental data on single-frequency oscillations in glycolysis qualitatively and which contains coupled first-order differential equations [38]. This system was then solved numerically by Mickens with the nonstandard finite difference scheme which preserves the property of positivity [39]. In the meantime, RDSs are usually used to describe certain realistic natural phenomena that can be involved in various processes such as neural networks, image processing, chemical reactions, and ecosystems. The presence of spatial variable means that such systems are frequently used in order to understand some irregular patterns such as self-replicating spikes, self-excitation, and spatio-temporal chaos. From this point of view and to further realize a wide variety of real-world problems, the importance of studying synchronization is highlighted for this model. In recent times, several considerable efforts have been made to examine the synchronization of RDSs, for example, in the bacterial cultures model [40], multi-layered natural networks [41], the FitzHugh–Nagumo system [42], and the Newton–Leipnik spatial-temporal chaotic system [43]. Moreover, appropriate controls have also been proposed for synchronizing different classes of partial differential equations (PDEs); see [44,45,46,47,48,49].

The present study concerns the analysis of the synchronization and control of the glycolysis model, which has the following general form:

(1) $$\left\{\begin{array}{cc} \frac{\partial u_{1}}{\partial t}=d_{1}\Delta u_{1}+f\left(t,u_{1},u_{2}\right) & \;\;x\in \;\Omega ,\;t>0\\ \frac{\partial u_{1}}{\partial t}=d_{2}\Delta u_{2}+g\left(t,u_{1},u_{2}\right) \end{array}\right.$$

where

$$f\left(t,u_{1},u_{2}\right)=bu_{2}-u_{1}+u_{1}^{2}u_{2}$$

$$g\left(t,u_{1},u_{2}\right)=a-bu_{2}-u_{1}^{2}u_{2}$$

and where $a,b,d_{1},d_{2}$ are positive constants, and $\Omega \subset R^{n}$ is a smoothly bounded domain with boundary ∂Ω, n≥1.

The free-diffusion model was the first original glycolysis model proposed by E.E. Selkov to describe the metabolic pathway that converts a type of sugar (glucose) into cellular energy (ATP). For a detailed background on the derivation and biochemical significance of model (1), we encourage the interested reader to consult the excellent overviews given in [50].

The glycolysis model (1) has been extensively studied in recent decades, but most research has been dedicated to the dynamics and the behavior of solutions including steady-state solutions, spatio-temporal periodic solutions, pattern formation, and global attractors [51,52,53,54]. Nevertheless, to the authors’ knowledge, this is the first study dealing with the synchronization and control of the RDS (1). This has motivated us to develop a suitable method to deal with the global synchronization of two glycolysis models. The remainder of this work is arranged in the following manner. In the next section, we discuss the existence and the uniform boundedness of the system’s solution, which will be surely useful for the ensuing parts. In Section 3, we design an appropriate control law in its linear form and furthermore prove the global asymptotic stability of the trivial solution associated with the error synchronization system, which consequently implies a global synchronization of a couple of systems that have the same form of system (1). Section 4 presents some applications and numerical simulations that demonstrate our findings. Finally, Section 5 is devoted to stating the conclusion of this work.

## 2. Problem Formulation

This section examines model (1) in view of two main aspects—the existence and the boundedness of its solution. Our analysis is based on a significant result associated with this type of system, which was obtained by Selwyn et al. in [55]. First, we assume that system (1) satisfies the following non-negative and uniformly bounded initial conditions:

(2) $$0\leq u_{1}\left(x,0\right),u_{2}\left(x,0\right)\leq M_{0},\;\mathrm{for}\;\mathrm{all}\;x\in \Omega ,\;\mathrm{where}\;M_{0}>0,$$

and the following homogeneous Neumann boundary conditions:

(3) $$\frac{\partial u_{1}}{\partial \nu }=\frac{\partial u_{2}}{\partial \nu }=0,\;\mathrm{for}\;\mathrm{all}\;x\in \partial \Omega ,\;t>0,$$

where ν is the unit vector normal to ∂Ω. One might observe that system (1) satisfies the following properties:

1. Based on proposition 1 in [55] and since $f,g:{\left[0,\infty \right)}^{3}\to R$ are continuous and differentiable functions in which f(t,0,η)≥0 and g(t,ξ,0)≥0, for all t,ξ,η≥0, we can deduce that system (1) has a local unique solution $\left(u_{1},u_{2}\right)$ on $\Omega \times \left[0,T^{*}\right)$, and furthermore there are two continuous functions $N_{1},N_{2}:\left[0,T^{*}\right)\to \left[0,\infty \right)$ such that:
  $$\begin{array}{cc} 0\leq u_{1}\left(x,t\right)\leq N_{1}\left(t\right),\;\;0\leq u_{2}\left(x,t\right)\leq N_{2}\left(t\right), & \;\mathrm{where}\;\left(x,t\right)\in \Omega \times \left[0,T^{*}\right). \end{array}$$
2. There is a constant γ≥1 and a continuous function $L_{0}:{\left[0,\infty \right)}^{2}\to \left[0,\infty \right)$ such that $\left|g(t,\xi ,\eta )\right|\leq L_{0}\left(t,r\right){\left(1+\eta \right)}^{\gamma }$, for all t,ξ,η≥0 with ξ≤r. This, consequently, implies:
  $$\left|g(t,\xi ,\eta )\right|\leq a+b\eta +\xi^{2}\eta \leq \left(a+b+\xi \right){\left(1+\eta \right)}^{2}.$$
3. There is a continuous function $\mu_{0}:{\left[0,\infty \right)}^{2}\to \left[0,\infty \right)$ so that $f\left(t,\xi ,\eta \right)+g\left(t,\xi ,\eta \right)\leq \mu_{0}\left(t,r\right)$, ∀t,ξ,η≥0 with ξ≤r. This, consequently, implies:
  f(t,ξ,η)+g(t,ξ,η)=a−ξ≤a.
4. The solution $u_{1}\left(x,t\right)$ is still uniformly bounded as a function of *t* in each bounded interval. To see this, one can refer to Lemma 2.2 in [54].

In fact, the aforementioned properties (1–4) can lead one to use Theorem 2, given in [55], and then to prove the next lemma.

**Lemma** **1.** *System (1) has a global continuous unique solution $\left(u_{1},u_{2}\right)$, which is uniformly bounded in Ω×[0,∞), and $\exists M\in R^{+}$ such that:*

$$\begin{array}{cc} 0\leq u_{1}\left(x,t\right),u_{2}\left(x,t\right)\leq M, & \;for\;all\;x\in \Omega \;and\;t>0. \end{array}$$

## 3. Synchronization

This section takes into consideration the drive-response formalism aiming to accomplish the synchronization of two coupled glycolysis systems. In this approach, we denote system (1) as the drive system and the other controlled system as the response system. Then, an appropriate controller is designed to force the errors of synchronization to converge to zero. The response system associated with system (1) can be given as follows:

(4) $$\left\{\begin{array}{cc} \frac{\partial v_{1}}{\partial t}=d_{1}\Delta v_{1}+f\left(t,v_{1},v_{2}\right)+U_{1},\; & x\in \;\Omega ,\;t>0\\ \frac{\partial v_{2}}{\partial t}=d_{2}\Delta v_{2}+g\left(t,v_{1},v_{2}\right)+U_{2},\; & x\in \;\Omega ,\;t>0\\ \frac{\partial v_{1}}{\partial \nu }=\frac{\partial v_{2}}{\partial \nu }=0\; & x\in \;\Omega ,\;t>0 \end{array}\right.$$

where $v_{i}=v_{i}\left(x,t\right),\;\left(i=1,2\right)$ are the states of system (4) and $U=(U_{1},U_{2})$ are the target controls that need to be adjusted immediately.

As we have mentioned before, one of the key objectives of this work is to design an appropriate control *U* for the purpose of forcing the error of synchronization $e\left(x,t\right)=(e_{1}\left(x,t\right),e_{2}\left(x,t\right))$ to converge to zero, in which this error can be defined via the differences between the states of system (1) and (4) as follows:

(5) $$\left(e_{1},e_{2}\right)=\left(v_{1}-u_{1},v_{2}-u_{2}\right).$$

In order to move forward to our next theoretical results, the definition below is stated for completeness.

**Definition** **1.** *The drive and response systems given, respectively, in (1) and (4) are considered to be globally synchronized if*

$$\lim_{t\to \infty }{∥e(x,t)∥}_{L^{2}}=0.$$

**Lemma** **2.** *There exists a positive constant K such that*

$$\left|v_{2}v_{1}^{2}-u_{2}u_{1}^{2}\right|\leq K\left(\left|v_{1}-u_{1}\right|+\left|v_{2}-u_{2}\right|\right).$$

**Proof.**  To begin with this proof, we first estimate the term $\left|v_{2}v_{1}^{2}-u_{2}u_{1}^{2}\right|$ as follows:

$$\begin{array}{ccc} \left|v_{2}v_{1}^{2}-u_{2}u_{1}^{2}\right| & \leq & \left|v_{2}v_{1}^{2}-u_{2}v_{1}^{2}\right|+\left|u_{2}v_{1}^{2}-u_{2}u_{1}^{2}\right|\\ & \leq & \left|v_{1}^{2}\right|\left|v_{2}-u_{2}\right|+\left|u_{2}\right|\left|v_{1}-u_{1}\right|\left|v_{1}+u_{1}\right|\\ & \leq & \left|v_{1}^{2}\right|\left|v_{2}-u_{2}\right|+\left|u_{2}\right|\left(\left|u_{1}\right|+\left|v_{1}\right|\right)\left|v_{1}-u_{1}\right|. \end{array}$$

Du to Lemma 1, we observe that the states $u_{1}$, $u_{2}$, and $v_{1}$ are uniformly bounded. Therefore, there exist three positive constants $K_{1}$, $K_{2}$, and $K_{3}$ such that:

$$\left|u_{1}\right|\leq K_{1},\left|u_{2}\right|\leq K_{2},\left|v_{1}\right|\leq K_{3}.$$

Thus, we have

$$\left|v_{2}v_{1}^{2}-u_{2}u_{1}^{2}\right|\leq K_{3}^{2}\left|v_{2}-u_{2}\right|+K_{2}\left(K_{1}+K_{3}\right)\left|v_{1}-u_{1}\right|.$$

To finish the proof, one can choose a constant *K* as follows:

$$K=\max\{K_{3}^{2},K_{2}\left(K_{1}+K_{3}\right)\},$$

and hence the desired result will be held.  □

**Theorem** **1.** *The drive and response systems given, respectively, in (1) and (4) are globally synchronized according to the control law:*

(6) $$\begin{array}{ccc} U_{1} & = & \left(2K+1\right)e_{1}-be_{2}, \end{array}$$

(7) $$\begin{array}{ccc} U_{2} & = & \left(2K+b\right)e_{2}. \end{array}$$

**Proof.**  Using notation (5), we can obtain the following error system:

(8) $$\left\{\begin{array}{cc} \frac{\partial e_{1}}{\partial t}=d_{1}\Delta e_{1}+be_{2}-e_{1}+v_{1}^{2}v_{2}-u_{1}^{2}u_{2}+U_{1}, & \;\mathrm{in}\;\Omega \times R^{+},\\ \frac{\partial e_{2}}{\partial t}=d_{2}\Delta e_{2}-be_{2}-v_{1}^{2}v_{2}+u_{1}^{2}u_{2}+U_{2}, & \mathrm{in}\;\Omega \times R^{+}. \end{array}\right.$$

Substituting the control law given in (6) and (7) into the above system yields:

(9) $$\left\{\begin{array}{cc} \frac{\partial e_{1}}{\partial t}=d_{1}\Delta e_{1}-2Ke_{1}+v_{1}^{2}v_{2}-u_{1}^{2}u_{2}, & \;\mathrm{in}\;\Omega \times R^{+},\\ \frac{\partial e_{2}}{\partial t}=d_{2}\Delta e_{2}-2Ke_{2}-v_{1}^{2}v_{2}+u_{1}^{2}u_{2}, & \;\mathrm{in}\;\Omega \times R^{+}, \end{array}\right.$$

which satisfies the zero Neumann boundary conditions. Now, we present our Lyapunov function as follows:

$$V=\frac{1}{2}\int_{\Omega }\left(e_{1}^{2}+e_{2}^{2}\right).$$

This exactly implies:

$$\frac{\partial V}{\partial t}=\int_{\Omega }\left(e_{1}\frac{\partial e_{1}}{\partial t}+e_{2}\frac{\partial e_{2}}{\partial t}\right)=\int_{\Omega }(d_{1}e_{1}\Delta e_{1}+d_{2}e_{2}\Delta e_{2}-2K\left(e_{1}^{2}+e_{2}^{2}\right)+\left(v_{1}^{2}v_{2}-u_{1}^{2}u_{2}\right)\left(e_{1}-e_{2}\right).$$

Consequently, using Green’s identity leads us to obtain the following assertion:

$$\begin{array}{cc} \frac{\partial V}{\partial t}\leq & -\int_{\Omega }d_{1}{\left|\nabla e_{1}\right|}^{2}dx+\int_{\partial \Omega }d_{1}e_{1}\frac{\partial e_{1}}{\partial \eta }d\sigma -\int_{\Omega }d_{2}{\left|\nabla e_{2}\right|}^{2}dx\\ \; & +\int_{\partial \Omega }d_{2}e_{2}\frac{\partial e_{2}}{\partial \eta }d\sigma -2K\int_{\Omega }\left(e_{1}^{2}+e_{2}^{2}\right)dx+\int_{\Omega }\left|v_{1}^{2}v_{2}-u_{1}^{2}u_{2}\right|\left|e_{1}-e_{2}\right|dx. \end{array}$$

With the help of Lemma 2, together with the Neumann boundary conditions, the term $\frac{\partial V}{\partial t}$ will be turned to be in the following estimation:

$$\begin{array}{cc} \frac{\partial V}{\partial t} & \leq -\int_{\Omega }d_{1}{\left|\nabla e_{1}\right|}^{2}dx-\int_{\Omega }d_{2}{\left|\nabla e_{2}\right|}^{2}dx-2K\int_{\Omega }\left(e_{1}^{2}+e_{2}^{2}\right)dx\\ \; & =K\left|e_{1}+e_{2}\right|\left|e_{1}-e_{2}\right|\\ \; & \leq -\int_{\Omega }\left[d_{1}{\left|\nabla e_{1}\right|}^{2}+d_{2}{\left|\nabla e_{2}\right|}^{2})\right]-2K\int_{\Omega }\left({\left|e_{1}\right|}^{2}+{\left|e_{2}\right|}^{2}\right)dx+K\int_{\Omega }{\left(\left|e_{1}\right|+\left|e_{2}\right|\right)}^{2}dx\\ \; & =-\int_{\Omega }\left[d_{1}{\left|\nabla e_{1}\right|}^{2}+d_{2}{\left|\nabla e_{2}\right|}^{2})\right]-K\int_{\Omega }{\left(\left|e_{1}\right|-\left|e_{2}\right|\right)}^{2}dx. \end{array}$$

That is;

$$\frac{\partial V}{\partial t}<0.$$

From the perspective of Lyapunov’s stability theory, which asserts the global asymptotic stability of the trivial solution to the error system (9), the drive system (1) and the response system (4) are globally synchronized, which completes the proof.  □

## 4. Numerical Simulations

In this section, we demonstrate some computational examples in one- and two-dimensional space to exemplify the practicability of the synchronization scheme proposed in this work. These simulations are carried out using some prepared codes in MATLAB based on the finite difference method (FDM), see [56,57] for a full overview of this scheme and how it could be implemented in synchronization problems. First of all, let us take x∈Ω=[0,10] with a step size equal to 0.2, t∈[0,100] with a step size equal to 4, $\left(d_{1},d_{2},a,b\right)=\left(0.01,1,3.5,0.25\right)$ and the initial conditions associated with the drive system (1) as follows:

(10) $$\left(u_{1}\left(x,0\right),u_{2}\left(x,0\right)\right)=\left(3.5+0.1\sin\left(x\right),0.28+0.1\sin\left(x\right)\right),$$

and the initial conditions associated with the response system (4) as follows:

(11) $$\left(v_{1}\left(x,0\right),v_{2}\left(x,0\right)\right)=\left(1+0.5\sin\left(0.2x\right),0.6+0.5\sin\left(0.2x\right)\right).$$

**Figure 1:**
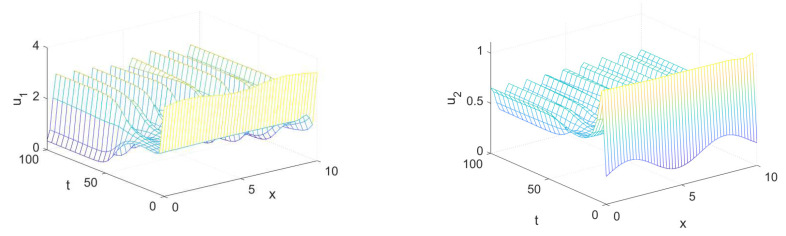
Dynamic behavior of the drive system (1) with $d_{1}=0.01$, $d_{2}=1$, a=3.5, and b=0.25 in accordance with the initial conditions given in (10).

**Figure 2:**
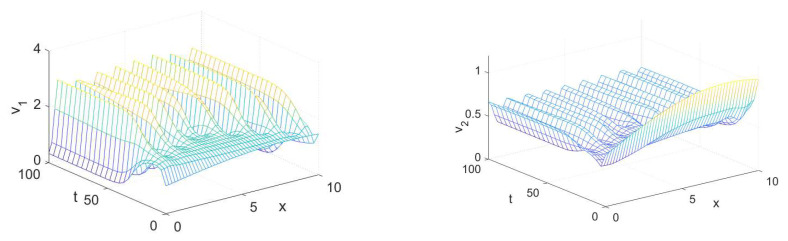
Dynamic behavior of the response system (4) with $d_{1}=0.01$, $d_{2}=1$, a=3.5, and b=0.25 in accordance with the initial conditions given in (11).

**Figure 3:**
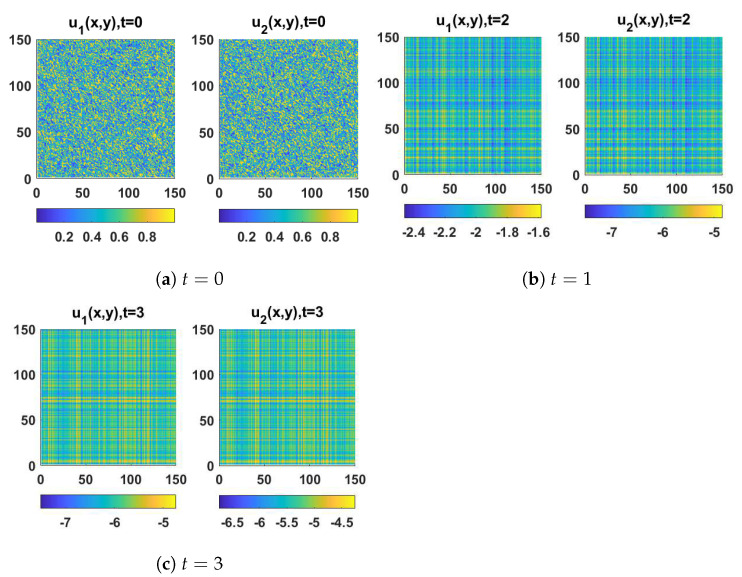
The solution of the drive system (1) in 2D space at (**a**) t=0, (**b**) t=1, and (**c**) t=3.

**Figure 4:**
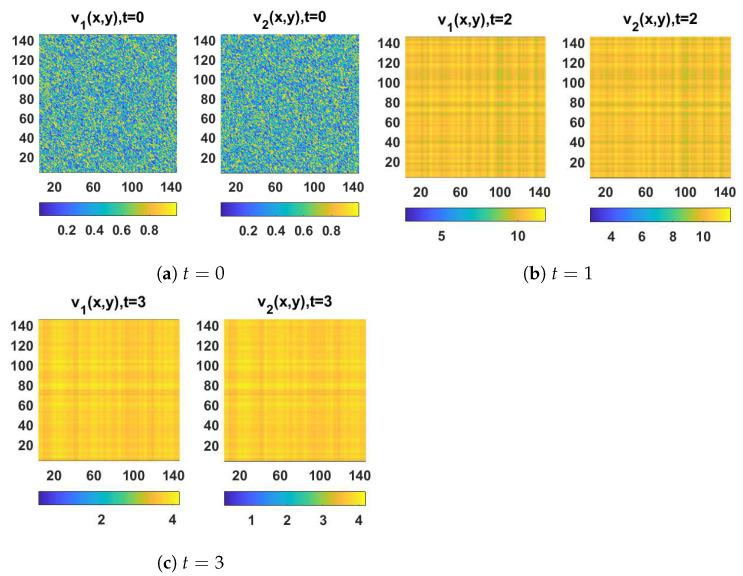
The solution of the response system (4) in 2D space at (**a**) t=0, (**b**) t=1, and (**c**) t=3.

**Figure 5:**
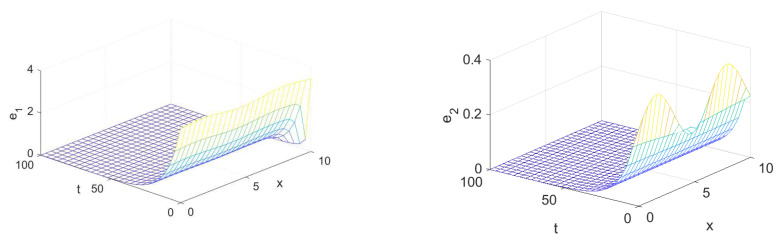
Dynamic behavior of the solutions of the spatiotemporal synchronization error system (5) with $d_{1}=0.01$, $d_{2}=1$, and K=0.2.

**Figure 6:**
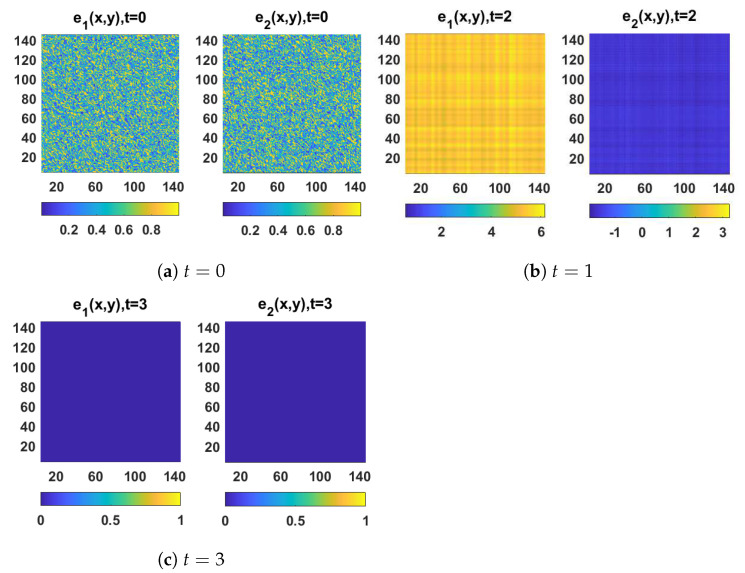
The solution of the spatiotemporal synchronization error system (5) in 2D-space at (**a**) t=0, (**b**) t=1, and (**c**) t=3.

The spatiotemporal solutions of system (1) and system (4) with homogeneous Neumann boundary conditions are depicted in Figure 1 and Figure 2, whereas Figure 3 and Figure 4 show the pattern formation associated with the two systems (1) and (4), respectively. In accordance with Theorem 1, if we choose $K=\frac{1}{5},$ then the two controllers $U_{1}$ and $U_{2}$ will be designed as follows:

$$\begin{array}{ccc} U_{1} & = & \frac{5}{7}\left(v_{1}-u_{1}\right)-b\left(v_{2}-u_{2}\right),\\ U_{2} & = & \frac{5b+2}{5}\left(v_{2}-u_{2}\right), \end{array}$$

and then system (1) and system (4) will be globally synchronized. To illustrate this numerically, the spatiotemporal solutions of the error synchronization system (5) are provided in Figure 5 and Figure 6 in one- and two-dimensional space. Indeed, this evolution clearly indicates that the errors converge to 0 as t→+∞.

## 5. Conclusions

For many years, many researchers have focused on the study of the synchronization of systems of ordinary differential equations and uni-dimensional maps. In the present work, we have developed an innovative approach to analyze the control synchronization of the nonlinear glycolysis spatiotemporal system. We first established the uniform boundedness of the solution, which was subsequently used in the implementation of the proposed control law. Then, we proved our findings rigorously using the Lyapunov direct method. Several numerical simulations have been illustrated to provide evidence of the efficacy and the performance of the established control approaches. In this regard, the simulation results have confirmed that the proposed control scheme is efficient for the purpose of synchronization. As a future research plan, we can focus on the use of optimal control techniques for the stabilization and synchronization of chaotic dynamical attractors employed in several applications, such as secure communications, applications for encryption, data sovereignty control, and many others.
